# Supplementary material for: In Vivo Effects of Balanced Crystalloid or Gelatine Infusions on Functional Parameters of Coagulation and Fibrinolysis: A Prospective Randomized Crossover Study
Source: J Pers Med. 2022 May 31;12(6):909. doi: 10.3390/jpm12060909 (PMC9225437; doi:10.3390/jpm12060909)
Supplement: Supplementary file 1 [file jpm-12-00909-s001.zip › jpm-1656541-supplementary.pdf]

Table S1. Description and reference values of standard laboratory tests[1] [48,49,50].

Standard Laboratory Test	Description	Clinical Interpretation	Reference Range Values
APTT [s]	Activated partial thromboplastin time	Measure of the integrity of the intrinsic and final common pathways of the coagulation cascade. Represents the time, in seconds, for patient plasma to clot after activation with contact pathway activator, while phospholipid and calcium ions suport assembly of tenase and prothrombinase complexes. Prolongation of the APTT results from deficiencies or inhibitors of clotting factors within the intrinsic and final common pathways.	25.4-36.9
D-dimers [ng/ml]	D-dimer concentration	degradation product of crosslinked (by factor XIIIa) fibrin. Reflects ongoing activation of the hemostatic system. by-product of the blood clotting and break-down proces.	<500
Fibrinogen [mg/dl]	Fibrinogen concentration	340kDa hexameric plasma glycoprotein synthesized by the liver. Has the maximum concentration amongst all the coagulation factors. Major structural component of a clot.	200-393
INR	International normalized ratio	The INR is a mathematical conversion of a patient's PT that accounts for the sensitivity of the thromboplastin used in a specific laboratory.	0.80-1.20
MPV [fl]	Mean platelet volume	Measure of the average size of platelets. Important in determining the cause of thrombocytopenia or thrombocytosis.	9.0-17.0
PDW [%]	Platelet distribution width	Reflects variability in platelet size, and is considered a marker of platelet function and activation.	9.0-13.0
P-LCR [%]	Platelet-large cell ratio	The percentage of platelets that exceed the normal value of platelet volume of 12 fL in the total platelet count.	13.0-43.0
PLT [10^3/µl]	Platelet count	Small fragments of megakaryocyte cells with numerous physiological purposes, including clotting activity and activation of the coagulation cascade. This activity is initiated with tissue injury and progresses into the release and binding of glycoproteins, growth factors, and clotting factors.	150-400

PT [s]	Prothrombin time	Measure of the integrity of the extrinsic and final common pathways of the procoagulant cascade. Represents the time, in seconds, for patient plasma to clot after the addition of calcium and an activation with thromboplastin. Prolongation of the PT results from deficiencies or inhibitors of clotting factors within the extrinsic and final common pathways.	9.4-12.5

[1]Measurements provided with the ACL TOP 300 CTS Hemostasis Testing System Analyser (Werfen Group Instrumentation Laboratory, Bedford, MA), using the HemosIL reagents: HemosIL APTT-SP, HemosIL Q.F.A. Thrombine (Bovine), HemosIL RecombiPlasTin 2G, HemosIL D-dimer HS 500

Table S2. Description and reference values of rotational thromboelastometry (ROTEM) parameters[1] [7,8].

**ROTEM Parameter**	**Description**	**Clinical Interpretation**	**Reference Range Values**
AA [◦]	reflects the kinetics of clot formation, defined as the angle between the baseline and a tangent to the clotting curve through the 2 mm point.	The decrease indicates platelet dysfunction or deficiency and fibrinogen deficiencies; increase may suggest hypercoagulability	EXTEM: 63-83<br>INTEM: 70-83
A10/A20 etc. [mm]	The amplitude of the clot firmness at a given point in time (in minutes)	Clot strength indicator at a given time during the assay run time, sometimes used as "transfusion triggers."	EXTEM A10: 43-65<br>INTEM A10: 44-66<br>FIBTEM A10: 7-23<br>EXTEM A20: 50-71<br>INTEM A20: 50-71<br>FIBTEM A20: 8-24
CFT [s]	indicates the time in seconds, between 2 and 20 mm clot firmness amplitude is achieved.	Represents the kinetics of clot formation. Indicates early the clot deficiency or state of hypercoagulability	EXTEM: 34-159<br>INTEM: 30-110

CT [s]	Time from the start of measurement till the formation of a 2 mm clot.	Prolongation may indicate a coagulation factors deficiency or presence of anticoagulants	EXTEM: 38-79<br>INTEM: 100-240<br>FIBTEM: 38-62
MCE [dynes/cm^2]	Maximum clot elasticity	A value related to the physical clot elasticity, which may assist in additional interpretation of MCF results.	EXTEM: 86-230<br>INTEM: 86-230
ΔMCE [-]	$\Delta MCE = MCE_{EXTEM} - MCE_{FIBTEM}$	Assessment of platelet contribution to clot strength. ΔMCE increases with platelet count.	
MCF [mm]	The maximum amplitude of the clot strength, influenced by fibrin, platelets, and factor XIII concentration	Indicates consistency or quality of the clot, characterizes the patient's coagulability state	EXTEM: 50-72<br>INTEM: 50-72<br>FIBTEM: 9-25
ML [%]	Maximum lysis detected during the run time, described in % of MCF	The clot is stable if the ML is less than 15%. Hyperfibrinolysis is indicated when greater than 15%.	EXTEM/INTEM <15%

[1]Abbreviations: A10, clot firmness amplitude measured after 10 minutes; A20, clot firmness amplitude measured after 20minutes; AA, alpha angle; CFT, clot forming time; CT, clotting time; MCF, maximum clot firmness; ML, maximum lysis.
